# Supplementary material for: Modeling glioblastoma heterogeneity as a dynamic network of cell states
Source: Mol Syst Biol. 2021 Sep 16;17(9):e10105. doi: 10.15252/msb.202010105 (PMC8444284; doi:10.15252/msb.202010105)
Supplement: Supplementary file 5 — Source Data for Figure 3 [file MSB-17-e10105-s001.zip › Figure3A_sourcedata/GSEA_3065/nosigdb_state5.GseaPreranked.1622718435914/NB_KI_CLUSTER3.html]

Details for gene set NB\_KI\_CLUSTER3[GSEA]

|  || Dataset | state5 |
| Phenotype | NoPhenotypeAvailable |
| Upregulated in class | na\_neg |
| GeneSet | NB\_KI\_CLUSTER3 |
| Enrichment Score (ES) | -0.5777243 |
| Normalized Enrichment Score (NES) | -2.0985646 |
| Nominal p-value | 0.0 |
| FDR q-value | 0.0 |
| FWER p-Value | 0.0 |
Table: GSEA Results Summary

  

Fig 1: Enrichment plot: NB\_KI\_CLUSTER3      
 Profile of the Running ES Score & Positions of GeneSet Members on the Rank Ordered List

  

| PROBE | GENE SYMBOL | GENE\_TITLE | RANK IN GENE LIST | RANK METRIC SCORE | RUNNING ES | CORE ENRICHMENT || 1 | HACD3 |  |  | 91 | 0.542 | 0.0028 | No |
| 2 | RTN3 |  |  | 123 | 0.505 | 0.0114 | No |
| 3 | SGCB |  |  | 328 | 0.370 | -0.0028 | No |
| 4 | ZNF738 |  |  | 399 | 0.342 | -0.0025 | No |
| 5 | ATP6V0B |  |  | 455 | 0.324 | -0.0009 | No |
| 6 | NSUN5 |  |  | 618 | 0.281 | -0.0125 | No |
| 7 | SFXN1 |  |  | 801 | 0.241 | -0.0273 | No |
| 8 | ZNF519 |  |  | 928 | 0.220 | -0.0363 | No |
| 9 | MMS22L |  |  | 1126 | 0.195 | -0.0539 | No |
| 10 | FADS1 |  |  | 1148 | 0.191 | -0.0517 | No |
| 11 | FSD1L |  |  | 1333 | 0.168 | -0.0685 | No |
| 12 | PPP1R35 |  |  | 1368 | 0.164 | -0.0684 | No |
| 13 | KNOP1 |  |  | 1445 | 0.157 | -0.0732 | No |
| 14 | COMMD2 |  |  | 1487 | 0.154 | -0.0742 | No |
| 15 | VPS45 |  |  | 1530 | 0.149 | -0.0754 | No |
| 16 | GPSM2 |  |  | 1612 | 0.141 | -0.0811 | No |
| 17 | SEC61A2 |  |  | 1684 | 0.135 | -0.0859 | No |
| 18 | FIGNL1 |  |  | 1696 | 0.133 | -0.0840 | No |
| 19 | SLC25A13 |  |  | 1707 | 0.133 | -0.0819 | No |
| 20 | CENPJ |  |  | 2032 | 0.109 | -0.1160 | No |
| 21 | DUS1L |  |  | 2120 | 0.104 | -0.1234 | No |
| 22 | CEP78 |  |  | 2121 | 0.104 | -0.1208 | No |
| 23 | SSBP2 |  |  | 2122 | 0.104 | -0.1183 | No |
| 24 | ASNS |  |  | 2215 | 0.097 | -0.1264 | No |
| 25 | CPSF3 |  |  | 2372 | 0.088 | -0.1420 | No |
| 26 | ZGRF1 |  |  | 2407 | 0.085 | -0.1438 | No |
| 27 | SYNC |  |  | 2430 | 0.083 | -0.1443 | No |
| 28 | POLE |  |  | 2438 | 0.083 | -0.1431 | No |
| 29 | KLHL42 |  |  | 2555 | 0.076 | -0.1544 | No |
| 30 | KLHL23 |  |  | 2595 | 0.074 | -0.1570 | No |
| 31 | ADAT1 |  |  | 2617 | 0.073 | -0.1577 | No |
| 32 | FANCL |  |  | 2649 | 0.071 | -0.1595 | No |
| 33 | HLTF |  |  | 2699 | 0.068 | -0.1634 | No |
| 34 | SCCPDH |  |  | 2799 | 0.063 | -0.1731 | No |
| 35 | CTDSPL2 |  |  | 2802 | 0.063 | -0.1718 | No |
| 36 | VKORC1L1 |  |  | 2945 | 0.055 | -0.1866 | No |
| 37 | DNAJC2 |  |  | 3148 | 0.044 | -0.2084 | No |
| 38 | CCP110 |  |  | 3202 | 0.041 | -0.2134 | No |
| 39 | ACTR6 |  |  | 3220 | 0.040 | -0.2144 | No |
| 40 | FLAD1 |  |  | 3282 | 0.037 | -0.2204 | No |
| 41 | WDR54 |  |  | 3602 | 0.023 | -0.2560 | No |
| 42 | RNF219 |  |  | 3709 | 0.020 | -0.2675 | No |
| 43 | MSH6 |  |  | 3736 | 0.019 | -0.2700 | No |
| 44 | METTL2A |  |  | 3767 | 0.017 | -0.2730 | No |
| 45 | PARP2 |  |  | 3831 | 0.014 | -0.2798 | No |
| 46 | PRR3 |  |  | 3847 | 0.014 | -0.2812 | No |
| 47 | TRIM24 |  |  | 3910 | 0.011 | -0.2879 | No |
| 48 | ZNF620 |  |  | 4135 | 0.002 | -0.3133 | No |
| 49 | FXR2 |  |  | 4273 | -0.005 | -0.3287 | No |
| 50 | HSF2 |  |  | 4351 | -0.008 | -0.3372 | No |
| 51 | MFAP1 |  |  | 4692 | -0.022 | -0.3752 | No |
| 52 | RRP7A |  |  | 4975 | -0.032 | -0.4064 | No |
| 53 | NLN |  |  | 5117 | -0.038 | -0.4215 | No |
| 54 | SLC25A17 |  |  | 5172 | -0.039 | -0.4267 | No |
| 55 | NUFIP1 |  |  | 5297 | -0.044 | -0.4397 | No |
| 56 | HIRIP3 |  |  | 5440 | -0.050 | -0.4545 | No |
| 57 | ACYP1 |  |  | 5479 | -0.052 | -0.4576 | No |
| 58 | CTCF |  |  | 5558 | -0.055 | -0.4651 | No |
| 59 | SINHCAF |  |  | 5822 | -0.066 | -0.4933 | No |
| 60 | TOPBP1 |  |  | 5920 | -0.070 | -0.5026 | No |
| 61 | MRPL44 |  |  | 6017 | -0.075 | -0.5117 | No |
| 62 | EXOSC8 |  |  | 6020 | -0.075 | -0.5101 | No |
| 63 | COPS7B |  |  | 6067 | -0.078 | -0.5134 | No |
| 64 | DCAKD |  |  | 6142 | -0.082 | -0.5199 | No |
| 65 | MANBAL |  |  | 6203 | -0.085 | -0.5246 | No |
| 66 | SNX4 |  |  | 6247 | -0.087 | -0.5274 | No |
| 67 | METAP2 |  |  | 6375 | -0.094 | -0.5395 | No |
| 68 | SH3GLB2 |  |  | 6381 | -0.094 | -0.5378 | No |
| 69 | GPS1 |  |  | 6457 | -0.099 | -0.5439 | No |
| 70 | UNG |  |  | 6479 | -0.100 | -0.5439 | No |
| 71 | GPN1 |  |  | 6673 | -0.112 | -0.5631 | No |
| 72 | NEK1 |  |  | 6679 | -0.112 | -0.5609 | No |
| 73 | INPP5F |  |  | 6724 | -0.115 | -0.5631 | No |
| 74 | GOT1 |  |  | 6759 | -0.118 | -0.5642 | No |
| 75 | DBNL |  |  | 6765 | -0.118 | -0.5619 | No |
| 76 | HPF1 |  |  | 6811 | -0.122 | -0.5640 | No |
| 77 | SPOP |  |  | 6824 | -0.123 | -0.5624 | No |
| 78 | NDUFAF6 |  |  | 6883 | -0.128 | -0.5659 | No |
| 79 | PRPSAP2 |  |  | 6905 | -0.129 | -0.5652 | No |
| 80 | WDR18 |  |  | 6908 | -0.130 | -0.5623 | No |
| 81 | SSX2IP |  |  | 6917 | -0.130 | -0.5601 | No |
| 82 | HSP90AA1 |  |  | 6962 | -0.134 | -0.5618 | No |
| 83 | CASP3 |  |  | 7082 | -0.144 | -0.5718 | No |
| 84 | MRPL10 |  |  | 7123 | -0.147 | -0.5728 | No |
| 85 | UTP18 |  |  | 7133 | -0.148 | -0.5703 | No |
| 86 | SEPHS2 |  |  | 7149 | -0.150 | -0.5684 | No |
| 87 | JPT2 |  |  | 7212 | -0.157 | -0.5716 | No |
| 88 | TRAP1 |  |  | 7267 | -0.163 | -0.5738 | Yes |
| 89 | EIF4E |  |  | 7292 | -0.166 | -0.5725 | Yes |
| 90 | FKBP4 |  |  | 7326 | -0.170 | -0.5721 | Yes |
| 91 | DLD |  |  | 7363 | -0.176 | -0.5720 | Yes |
| 92 | VPS72 |  |  | 7384 | -0.179 | -0.5699 | Yes |
| 93 | DDX1 |  |  | 7395 | -0.180 | -0.5667 | Yes |
| 94 | CISD1 |  |  | 7408 | -0.182 | -0.5637 | Yes |
| 95 | ENOPH1 |  |  | 7445 | -0.186 | -0.5633 | Yes |
| 96 | LCMT1 |  |  | 7468 | -0.188 | -0.5613 | Yes |
| 97 | MAGOHB |  |  | 7542 | -0.196 | -0.5648 | Yes |
| 98 | GARS |  |  | 7583 | -0.202 | -0.5645 | Yes |
| 99 | ACOT7 |  |  | 7623 | -0.207 | -0.5639 | Yes |
| 100 | FAM104A |  |  | 7663 | -0.213 | -0.5632 | Yes |
| 101 | CARHSP1 |  |  | 7667 | -0.213 | -0.5584 | Yes |
| 102 | BAZ1B |  |  | 7669 | -0.213 | -0.5534 | Yes |
| 103 | GIHCG |  |  | 7706 | -0.220 | -0.5522 | Yes |
| 104 | HDGFL2 |  |  | 7792 | -0.233 | -0.5562 | Yes |
| 105 | RFK |  |  | 7798 | -0.234 | -0.5512 | Yes |
| 106 | TAF9 |  |  | 7817 | -0.238 | -0.5475 | Yes |
| 107 | TUBG1 |  |  | 7846 | -0.243 | -0.5448 | Yes |
| 108 | CCDC25 |  |  | 7855 | -0.244 | -0.5398 | Yes |
| 109 | NELFE |  |  | 7882 | -0.251 | -0.5367 | Yes |
| 110 | NDUFA4 |  |  | 7910 | -0.257 | -0.5336 | Yes |
| 111 | CDKN2C |  |  | 8091 | -0.295 | -0.5469 | Yes |
| 112 | SLC25A39 |  |  | 8115 | -0.299 | -0.5423 | Yes |
| 113 | SLC25A4 |  |  | 8128 | -0.303 | -0.5363 | Yes |
| 114 | PSMB4 |  |  | 8147 | -0.308 | -0.5310 | Yes |
| 115 | PSMC4 |  |  | 8153 | -0.309 | -0.5241 | Yes |
| 116 | PRMT1 |  |  | 8185 | -0.321 | -0.5199 | Yes |
| 117 | HMGN1 |  |  | 8192 | -0.324 | -0.5127 | Yes |
| 118 | SUCLG1 |  |  | 8202 | -0.327 | -0.5059 | Yes |
| 119 | IFT22 |  |  | 8246 | -0.337 | -0.5026 | Yes |
| 120 | CCDC167 |  |  | 8260 | -0.342 | -0.4959 | Yes |
| 121 | EIF3I |  |  | 8290 | -0.351 | -0.4907 | Yes |
| 122 | EIF4EBP1 |  |  | 8306 | -0.356 | -0.4838 | Yes |
| 123 | SNRPC |  |  | 8316 | -0.359 | -0.4762 | Yes |
| 124 | HSPA8 |  |  | 8339 | -0.366 | -0.4699 | Yes |
| 125 | ARPC5L |  |  | 8371 | -0.379 | -0.4642 | Yes |
| 126 | LDHB |  |  | 8372 | -0.379 | -0.4551 | Yes |
| 127 | HSPD1 |  |  | 8418 | -0.393 | -0.4507 | Yes |
| 128 | MRPL33 |  |  | 8467 | -0.417 | -0.4461 | Yes |
| 129 | EIF5A |  |  | 8468 | -0.418 | -0.4361 | Yes |
| 130 | CACYBP |  |  | 8486 | -0.428 | -0.4277 | Yes |
| 131 | COA3 |  |  | 8521 | -0.444 | -0.4208 | Yes |
| 132 | NDUFAF8 |  |  | 8527 | -0.446 | -0.4106 | Yes |
| 133 | MRPL17 |  |  | 8528 | -0.446 | -0.3999 | Yes |
| 134 | NT5DC2 |  |  | 8531 | -0.447 | -0.3893 | Yes |
| 135 | COPRS |  |  | 8615 | -0.502 | -0.3866 | Yes |
| 136 | HRAS |  |  | 8633 | -0.514 | -0.3762 | Yes |
| 137 | CYCS |  |  | 8640 | -0.519 | -0.3644 | Yes |
| 138 | COX5B |  |  | 8646 | -0.523 | -0.3523 | Yes |
| 139 | ARHGDIA |  |  | 8659 | -0.531 | -0.3409 | Yes |
| 140 | CAMTA1 |  |  | 8664 | -0.535 | -0.3284 | Yes |
| 141 | HMGA1 |  |  | 8675 | -0.543 | -0.3165 | Yes |
| 142 | UBE2M |  |  | 8690 | -0.551 | -0.3048 | Yes |
| 143 | PGAM1 |  |  | 8698 | -0.558 | -0.2922 | Yes |
| 144 | SRP14 |  |  | 8701 | -0.565 | -0.2788 | Yes |
| 145 | AKR1B1 |  |  | 8727 | -0.588 | -0.2674 | Yes |
| 146 | ENO1 |  |  | 8734 | -0.593 | -0.2538 | Yes |
| 147 | UBB |  |  | 8745 | -0.602 | -0.2405 | Yes |
| 148 | ANAPC11 |  |  | 8773 | -0.630 | -0.2283 | Yes |
| 149 | SET |  |  | 8813 | -0.664 | -0.2168 | Yes |
| 150 | FABP5 |  |  | 8829 | -0.684 | -0.2020 | Yes |
| 151 | ODC1 |  |  | 8839 | -0.697 | -0.1862 | Yes |
| 152 | ACTG1 |  |  | 8859 | -0.718 | -0.1711 | Yes |
| 153 | RPL37A |  |  | 8860 | -0.721 | -0.1537 | Yes |
| 154 | ARPC1A |  |  | 8879 | -0.744 | -0.1378 | Yes |
| 155 | PPP1R14B |  |  | 8900 | -0.771 | -0.1215 | Yes |
| 156 | RAC1 |  |  | 8919 | -0.815 | -0.1039 | Yes |
| 157 | TPI1 |  |  | 8939 | -0.852 | -0.0855 | Yes |
| 158 | YBX1 |  |  | 8948 | -0.872 | -0.0654 | Yes |
| 159 | TXN |  |  | 8952 | -0.885 | -0.0445 | Yes |
| 160 | PKM |  |  | 8959 | -0.915 | -0.0231 | Yes |
| 161 | CFL1 |  |  | 8978 | -1.071 | 0.0007 | Yes |
Table: GSEA details [plain text format]

  

Fig 2: NB\_KI\_CLUSTER3: Random ES distribution      
 Gene set null distribution of ES for **NB\_KI\_CLUSTER3**
